# Late‐onset plate‐like osteoma cutis: Dermoscopic, histopathological, and ultrasound features

**DOI:** 10.1111/srt.13510

**Published:** 2023-10-25

**Authors:** Chiara Benaglia, Eleonora Di Michele, Angelo Valerio Marzano, Emanuela Passoni, Gianluca Nazzaro

**Affiliations:** ^1^ Dipartimento di Fisiopatologia Medico‐Chirurgica e dei Trapianti Università degli Studi di Milano Milan Italy; ^2^ SC Dermatologia Fondazione IRCCS Ca’ Granda Ospedale Maggiore Policlinico Milan Italy

Osteoma cutis (OC) is an aberrant development or deposit of mature lamellar bone within dermis and subcutaneous tissue. We report a case of acquired plate‐like OC on the scalp, focusing on dermoscopic and ultrasound features.

A 23‐year‐old man presented with an irregular plaque localized at the left fronto‐temporal area since the age of 14, arising as a small verrucous neoformation and with significant growth over time. The patient had no concomitant disease and was not taking any therapy.

On clinical examination, a 7 × 3 cm pinkish plaque with yellowish keratotic plugs and papular satellite lesions extending toward the left eyebrow, was present; the lesion had hard‐ligneous consistency and was associated with alopecia (Figure 1A). It was asymptomatic; however, caused a significant subjective cosmetic discomfort. On dermoscopy a reddish plaque, characterized by linear white and pink lines, was noticed. Keratotic plugs filled by amorphous white and yellow material were also detectable (Figure 2A,B). Skin ultrasound showed linear hyperechoic dermal band characterized by anechoic shadow cone, thus suggesting the presence of calcific materials (Figure 3).

**FIGURE 1:**
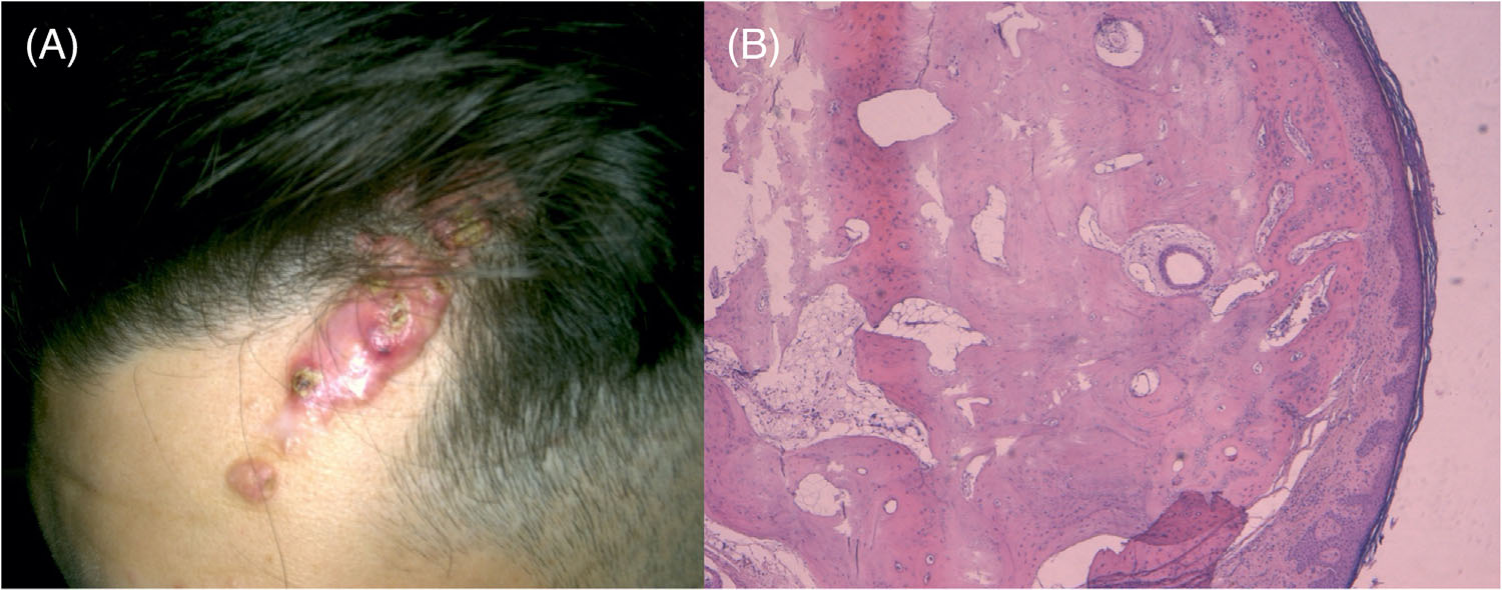
(A) Clinical features: a pinkish 7 × 3 cm plaque associated with papular satellite lesions extending toward the left eyebrow; associated with alopecia. (B) Histopathology (H &E 2x): the histology revealed a normal orthokeratotic epithelium while fragments of mature lamellar bone are localized at the level of the reticular dermis.

**FIGURE 2:**
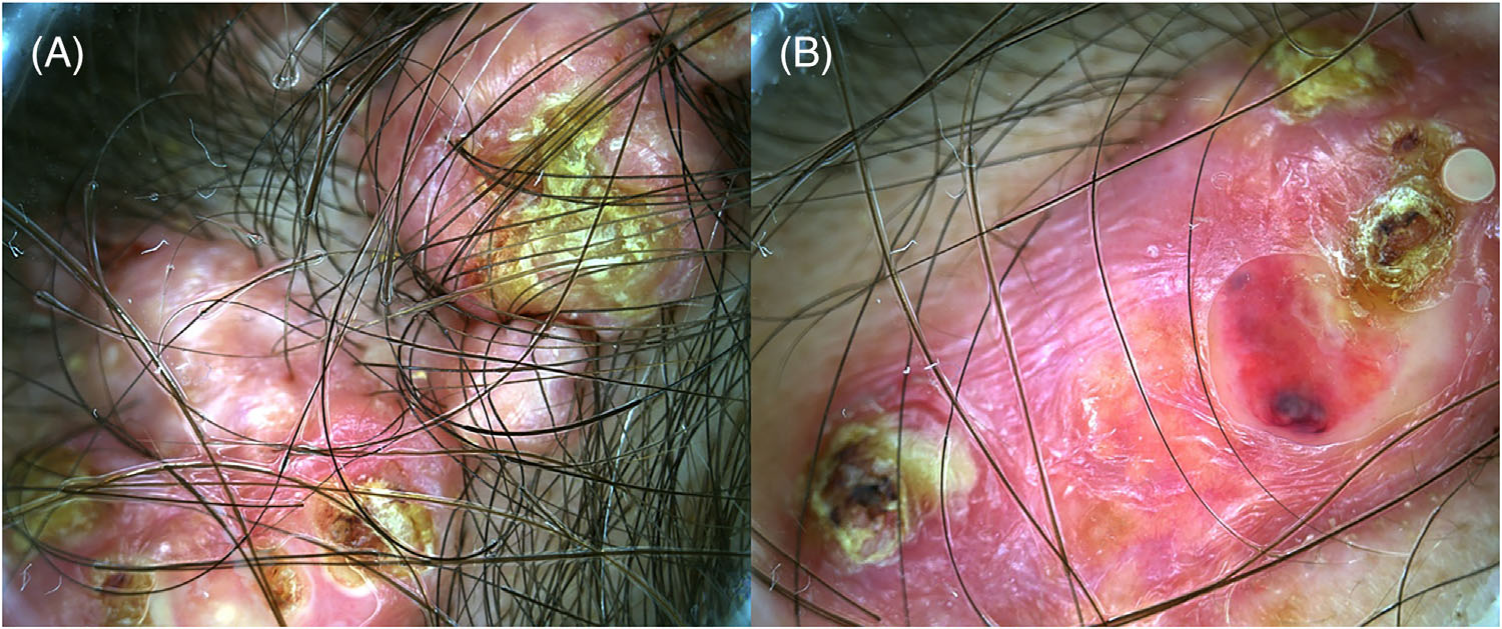
(A, B) Dermatoscopic image: erythematous plaque characterized by whitish streaks (milk appearance) surrounding yellowish amorphous plugs.

**FIGURE 3:**
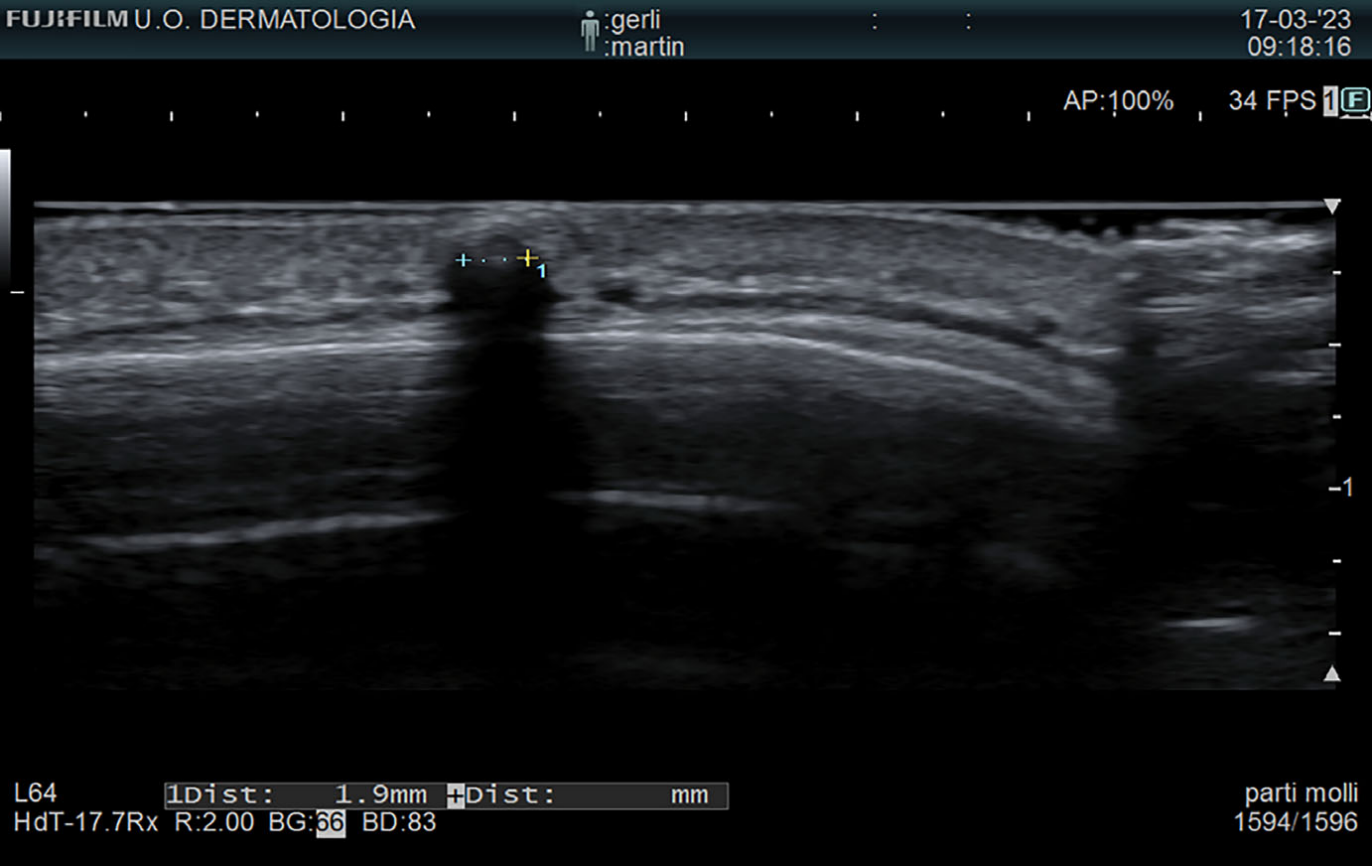
High‐frequency sonography [15–18 MHz]: hyperechoic reflective roundish structures associated with hypoechoic shadow cone.

Histology revealed bone deposits surrounded by plasma cells, granulocytes and neutrophils at the medium and deep dermis (Figure 1B).

Blood tests ruled out metabolic disorders or abnormalities in calcium and phosphorus; a skull X‐ray demonstrated no involvement of the cranial theca.

A diagnosis of plate‐like OC was performed, basing on histologic findings and the presence of all diagnostic criteria, except for the early onset.^1^

The patient refused complete excision due to possible cosmetic consequences resulting from a wide surgical scar. He decided to remove only a periocular papule because of visual discomfort.

Primary OC is a rare benign condition, first described by Wilkens in 1858, as an aberrant presence of bone in the dermis or hypodermis.^2^

The classification of OC distinguishes two types: primary and secondary. Primary OC is a rare condition, about 15% of all skin ossifications, corresponding to *de novo* bone formation, and it can be associated to syndromic diseases such as Albright's hereditary osteodystrophy (AHO), progressive ossificans fibrodysplasia (POF), or progressive osseus heteroplasia (POH).^3^ Secondary OC, instead, has been described in association with inflammatory diseases such as scleroderma or dermatomyositis, neoplastic ones such as ossified pigmented naevus, basal cell carcinoma and pilomatrixoma, or following traumatic events.

Clinical classification of OC distinguishes four patterns of presentation: single, plate‐like, widespread, or multiple miliary. The term *osteoma cutis in plaque* was coined by Worret and Burgdorf in 1978.^2^ This rare entity needs to fulfill the following features: presence since birth or in the first year of life; no association with metabolic disorders; presence or not of other associated osteomas; lack of trauma, infections or other predisposing factors.^1^ Although these criteria, there are also a few reports of acquired primary OC presenting mainly during the second and third decade, as our patient.^1^ Plate‐like OCs that appear during adult life are more frequent in men, and the most common location is the head.^4^ The pathogenesis of OC is unclear, mutations in the gene GNAS1 could play a role in AHO, POH and in congenital plate‐like OC,^5^ but the same mutations have not been identified in acquired plaque‐like OC.

Although histopathology remains the gold standard the diagnosis, we want to draw attention to non‐invasive tools, such as ultrasound and dermoscopy. Only two sonographic reports of OC are present in literature:^6^ hyperechoic structures associated by hypoechoic shadow suggest the calcific nature of the lesion.

Dermoscopy features of OC also have been rarely reported: the milkish appearance, corresponding to a yellowish amorphous area with whitish streaks, suggests the ossification.^7^, ^8^

Plate‐like OC is not associated with risk of neoplastic transformation; therefore, the treatment should consider the patient's wishes regarding the cosmetic outcome.

## CONFLICT OF INTEREST STATEMENT

The authors declare no conflicts of interest.

## Data Availability

Not applicable.
